# Three years of growth hormone therapy in children born small for gestational age: results from the ANSWER Program

**DOI:** 10.1530/EC-18-0286

**Published:** 2018-08-23

**Authors:** Robert Rapaport, Peter A Lee, Judith L Ross, Paul Saenger, Vlady Ostrow, Giuseppe Piccoli

**Affiliations:** 1 Ichan School of MedicineNew York, New York, USA; 2 Penn State College of MedicineHershey, Pennsylvania, USA; 3 Thomas Jefferson UniversityPhiladelphia, Pennsylvania, USA; 4 Nemours/DuPont Hospital for ChildrenWilmington, Delaware, USA; 5 Winthrop University HospitalMineola, New York, USA; 6 Novo Nordisk Inc.Plainsboro, New Jersey, USA

**Keywords:** small for gestational age (SGA), growth disorders, growth hormone therapy, height standard deviation score, growth hormone status

## Abstract

Growth hormone (GH) is used to treat short stature and growth failure associated with growth disorders. Birth size and GH status variably modulate response to GH therapy. The aim of this study was to determine the effect of birth size on response to GH therapy, and to determine the impact of GH status in patients born small for gestational age (SGA) on response to GH therapy. Data from the prospective, non-interventional American Norditropin Studies: Web-Enabled Research (ANSWER) Program was analyzed for several growth outcomes in response to GH therapy over 3 years. GH-naïve children from the ANSWER Program were included in this analysis: SGA with peak GH ≥10 ng/mL (20 mIU/L), SGA with peak GH <10 ng/mL (20 mIU/L), isolated growth hormone deficiency (IGHD) born SGA, IGHD not born SGA and idiopathic short stature. For patients with IGHD, those who did not meet criteria for SGA at birth showed greater improvements in height SDS and BMI SDS than patients with IGHD who met criteria for SGA at birth. For patients born SGA, response to GH therapy varied with GH status. Therefore, unlike previous guidelines, we recommend that GH status be established in patients born SGA to optimize GH therapy.

## Introduction

Treatment with recombinant human growth hormone (GH) is widely utilized for improving height in children with growth failure and conditions in which it is efficacious, including isolated growth hormone deficiency (IGHD), idiopathic short stature (ISS) and small for gestational age (SGA) (1, 2). Although clinical characteristics of these growth disorders often overlap, criteria for SGA can be distinguished from those of other GH disorders in that diagnosis is defined by having a birth weight and/or length of <−2.0 standard deviation scores (SDS) for gestational age (3). Retrospective studies indicate that approximately one-quarter of children with short stature (4) and one-third of children with IGHD (5) were reported to have historical auxological data consistent with being born SGA.

The effectiveness of GH therapy for increasing short-term growth rates and adult height in patients with various growth disorders is well documented (6, 7, 8, 9, 10, 11). For example, 2 years of GH treatment resulted in an increase from baseline in height SDS (HSDS) in children with Noonan syndrome, Turner syndrome, ISS, SGA, IGHD and multiple pituitary hormone deficiency (MPHD) (12, 13). Additional studies have also shown improved growth outcomes in these disorders after 4 to 5 years of GH treatment (7, 9, 14, 15) and improved growth outcomes associated with starting GH therapy at an earlier age in prepubertal children and for a longer duration (9, 12, 13, 15). Despite these demonstrated benefits of GH replacement therapy (6, 8, 10, 11), response to treatment with GH is substantially variable among diagnostic categories and among individuals with the same disorder (16, 17). For example, greater linear growth in response to 2 years of GH therapy was seen in children with SGA compared with those with IGHD (6). Several factors, including genetics, diagnosis and biochemical variability, contribute to differences in responsiveness to GH therapy (18) and complicate treatment decisions regarding GH therapy, such as when to start treatment and what dosage is appropriate. In an attempt to explain variability in response to GH therapy, several mathematical models have been developed as practical tools to estimate the response to GH therapy for the diagnosis of growth hormone deficiency (GHD) (19), SGA (20) and ISS (21).

The non-interventional American Norditropin Studies: Web-Enabled Research (ANSWER) Program was launched in 2002 and designed to assess the real-life clinical outcomes of pediatric and adult patients treated with Norditropin as prescribed by physicians according to standard clinical practice (22). Prospective collection of data through the US-based ANSWER Program offered the possibility to gather new insights into GH treatment effects in specific diagnostic populations, with respect to patient characteristics, diagnosis, age, sex and pubertal status. Previous publications from the ANSWER Program have shown that treatment with GH was efficacious in increasing HSDS from baseline in children with IGHD, MPHD, ISS, Noonan syndrome and Turner syndrome (7, 13). Additionally, some reports have investigated the effects of baseline characteristics (eg, HSDS, age, insulin-like growth factor 1 (IGF-I) SDS) on changes in HSDS in response to GH therapy over time (12, 13). The analysis described here evaluated growth outcomes (HSDS, IGF-I SDS and BMI SDS), bone age per chronological age (BA/CA) and GH dose over 3 years of treatment in the IGHD, ISS and SGA (GH-sufficient or -deficient) pediatric populations. The objective of this report is to assess whether GH responses in patients born SGA are affected by GH sufficiency/deficiency as indicated by GH stimulation tests and to evaluate the effect of birth size on response to GH therapy in patients with IGHD.

## Patients and methods

### Study design

Data were extracted from the prospective, non-interventional ANSWER Program, which was launched in 2002 and aimed to evaluate long-term safety and effectiveness outcomes of US pediatric and adult patients treated with Norditropin (somatropin (rDNA origin) injection; Novo Nordisk A/S) (23). The ANSWER Program enrolled adults and children prescribed Norditropin for treatment of GHD or other growth disorders. Informed consent from patients or their parents or guardians and approval by the appropriate institutional review board were obtained prior to study enrollment. Participating physicians recorded patient histories and physical examination data using the NovoNet Web-based research platform. Data collected at initial patient visits included baseline HSDS, weight, bone age, maximal stimulated serum GH concentration and serum IGF-I levels. Patient information collected at follow-up visits included GH dose/frequency, height, weight, IGF-I concentration and bone age. GH doses were determined by the treating physicians (22). Study protocols and documentation were approved by institutional review boards at each clinical site, and patients were required to provide informed consent before the start of any study-related activities. This study was conducted in accordance with the Declaration of Helsinki, and all data are anonymized. The ANSWER Program is guided by Good Pharmacoepidemiology Practice guidelines for design and reporting of epidemiologic studies, as defined by the International Society for Pharmacoepidemiology and Strengthening the Reporting of Observational studies in Epidemiology guidelines (22).

### Analysis population

The analyses of data from the ANSWER Program described in this report evaluated changes in growth-related clinical end points in response to 3 years of treatment with Norditropin. The analysis population included GH treatment-naïve children (aged <18 years at the end of 3 years of data collection) diagnosed with ISS, IGHD or SGA (defined as having a birth weight and/or body length SDS of <−2.0 for gestational age). ISS was defined as idiopathic short stature when excluding all other potential causes of growth failure. Diagnostic classification was determined by the participating physician who evaluated the patient. The IGHD population was subdivided based on fulfillment of SGA criteria according to historical auxological data, and the SGA population was subdivided by peak GH levels at baseline (<10 vs ≥10 ng/mL, or <20 vs ≥20 mIU/L) as determined by GH stimulation test. This cutoff point of 10 ng/mL (20 mIU/L) is commonly considered a threshold indicating normal or below normal GH levels (1); therefore, for the SGA population, a level of ≥10 ng/mL (20 mIU/L) was considered indicative of GH sufficiency, while a peak GH level of <10 ng/mL (20 mIU/L) was considered to indicate GH deficiency. In the ISS cohort, no patients had historical auxological data that fulfilled SGA criteria. Subpopulations are mutually exclusive; patients with IGHD born SGA were not included in SGA groups.

Only patients who were Tanner stage 1 (prepubertal) at baseline were included. Patients with prior GH treatment who started more than 1 week prior to enrollment were excluded. We also excluded patients with missing birth weight and/or length information at baseline (enrollment visit). Participants with missing, inconsistent or improbable baseline values of key variables (height, BMI, BA, CA, gender and IGF-I) or any of the GH response end points above were also excluded. Since many patients were missing baseline GH peak values and/or IGF-I values and/or BA imaging values at baseline, we used in lieu of missing any of these three baseline values the values from the closest visit date to enrollment date within the interval from 18 months before to 12 months after the enrollment date.

### Variables and statistical analysis

Clinical end points included HSDS, BMI SDS, bone age per chronological age (BA/CA), insulin-like growth factor 1 (IGF-I) SDS and GH dose. HSDS and BMI SDS (*z*-scores) were calculated from actual height and weight using standard references provided by the US Centers for Disease Control and Prevention (24). IGF-I values were measured locally and transformed into IGF-I SDS based on age- and sex-related normative values of Brabant *et al*. (25). Reported means, including those referring to change over baseline values, were calculated as a mean of the patients within each subpopulation. Patients with missing birth weight and/or length information or with missing baseline values of key variables (height, BMI, BA, CA, gender and IGF-I) or any of the clinical end points were excluded. Only observed values were used in the analysis; missing values were not imputed. Participants with missing data for some, but not all, the follow-up visits were included for the years with available data.

All statistical tests were two-sided and *P* values ≤0.050 were considered statistically significant. Descriptive summaries were provided for baseline characteristics and clinical end points at each year of GH treatment and reported as mean (standard deviation (s.d.)) unless otherwise noted. Differences in ΔHSDS and ΔIGF-I SDS at each year of GH treatment were compared by analysis of covariance (ANCOVA), with diagnostic population, gender and baseline age as fixed effect covariates. Least-squares (LS) means for ΔHSDS and ΔIGF-I SDS at year 3 were estimated using the Tukey–Kramer test. All analyses were performed using SAS version 9.3 (SAS Institute).

## Results

### Demographics and baseline characteristics by SGA, IGHD subpopulations

Of the 14,680 GH-naïve children who enrolled in ANSWER by the end of July 2016, 882 met all inclusion criteria and were included in this analysis. The subpopulations in this analysis were SGA and GH sufficient (peak GH ≥10 ng/mL, or ≥20 mIU/L), SGA with GH deficiency (peak GH <10 ng/mL, or <20 mIU/L), IGHD born SGA, IGHD not born SGA and ISS. The majority of these patients were diagnosed with IGHD and not born SGA (*n* = 709); the second largest subpopulation was those diagnosed with IGHD who were also born SGA (*n* = 86) (Table 1). Mean BA/CA was not statistically different among subpopulations, although statistical differences in means for all other parameters were found. Mean (s.d.) peak GH (ng/mL) levels were higher for children born SGA who were GH sufficient (17.04 (5.08)) compared to those born SGA who were GH deficient. For children with IGHD, those who met criteria for SGA had lower mean (s.d.) peak GH (ng/mL) levels compared to those with IGHD who did not meet criteria for SGA (6.82 (5.30) vs 7.44 (7.11)). Mean (s.d.) birth length SDS (BL SDS) was lower for children born SGA who were GH sufficient (−2.75 (1.97)) compared to those born SGA who were GH deficient (−2.08 (1.35)). Mean (s.d.) birth weight SDS (BW SDS) was lower for children born SGA who were GH sufficient (−2.50 (0.72)) compared to children born SGA with GH deficiency (−1.89 (1.12)).

**Table 1:** Demographics and baseline characteristics by reported diagnoses^a^.

	SGA, peak GH ≥10 ng/mL	SGA (non-IGHD), peak GH <10 ng/mL	IGHD born SGA	IGHD not born SGA	ISS	*P* value
*n*	28	15	86	709	44	
Male, *n* (%)	18 (64.3)	6 (40.0)	58 (67.4)	578 (81.5)	37 (84.1)	<0.001
Age, mean (s.d.) (years)	7.18 (3.40)	7.71 (2.75)	8.32 (3.30)	9.80 (2.96)	10.41 (2.35)	<0.001
Mean (s.d.)						
IGF-I SDS	−1.51 (1.34)	−1.06 (1.66)	−1.22 (1.51)	−1.88 (1.55)	−0.65 (2.08)	<0.001
BA/CA	0.78 (0.18)	0.79 (0.18)	0.83 (0.19)	0.83 (0.16)	0.85 (0.12)	0.43
BMI SDS	−1.07 (1.51)	−0.72 (2.06)	−0.41 (1.26)	−0.24 (1.28)	−0.31 (1.06)	0.008
Peak GH (ng/mL)	17.04 (5.08)	5.72 (2.84)	6.82 (5.30)	7.44 (7.11)	14.61 (3.95)	<0.001
Height SDS	−2.78 (0.71)	−2.29 (0.60)	−2.56 (0.76)	−2.25 (0.78)	−2.13 (0.46)	<0.001
BL SDS	−2.75 (1.97)	−2.08 (1.35)	−3.67 (3.25)	0.07 (1.01)	−0.35 (0.80)	<0.001
BW SDS	−2.50 (0.72)	−1.89 (1.12)	−2.48 (0.91)	−0.37 (1.08)	−0.68 (0.69)	<0.001
GH dose (mg/kg/day)	0.05 (0.02)	0.05 (0.02)	0.05 (0.01)	0.05 (0.01)	0.06 (0.02)	<0.001

^a^Subpopulations are mutually exclusive; patients with IGHD born SGA were not included in SGA groups.

BA/CA, bone age/chronological age; BL, birth length; BMI, body mass index; BW, birth weight; GH, growth hormone; IGF-I, insulin-like growth factor 1; IGHD, isolated growth hormone deficiency; ISS, idiopathic short stature; s.d., standard deviation; SDS, standard deviation score; SGA, small for gestational age.

### GH response and treatment summaries by year after enrollment and subpopulation

In general, the growth-related outcomes, IGF-I SDS, BMI SDS and HSDS improved over the 3 years of follow-up, as shown by mean SDS for these outcomes at each year after enrollment (Table 2). For all five patient groups, BA/CA increased slightly from year 1 to year 3. Among patients born SGA, mean (s.d.) BMI SDS from year 1 to year 3 increased more for those with GH deficiency (−0.85 (2.28) to −0.21 (1.11)) compared with those who were GH sufficient (year 1, −0.90 (1.43); year 3, −0.57 (1.06)). However, differences in mean (s.d.) height SDS from year 1 to year 3 were greater for patients born SGA who were GH sufficient (year 1, −2.43 (0.71); year 3, −1.63 (0.73)) compared to patients born SGA who were GH deficient (year 1, −1.89 (0.60); year 3, −1.18 (0.79)). Increase in mean (s.d.) IGF-I SDS was also greater for patients born SGA with GH deficiency (year 1, −0.11 (1.46); year 3, 1.19 (2.29)) compared to patients born SGA who were GH sufficient (year 1, 0.68 (2.17); year 3, 1.40 (1.14)). Improvements in growth-related outcomes were greater for IGHD patients who were not born SGA compared to those with IGHD who were born SGA. Increases in mean (s.d.) HSDS from year 1 to year 3 were slightly greater for patients with IGHD not born SGA (−1.86 (0.78) to −1.04 (0.80)) than for patients with IGHD born SGA (−2.08 (0.69) to −1.34 (0.72)). Similarly, increase in mean (s.d.) BMI SDS was greater for IGHD patients not born SGA (year 1, −0.18 (1.10); year 3, 0.02 (1.07)) compared to IGHD patients born SGA (year 1, −0.43 (1.16); year 3, −0.28 (1.03)). Of note, mean (s.d.) IGF-I SDS increased from 0.19 (1.69) to 1.00 (1.54) from year 1 to year 3 for IGHD patients not born SGA compared to an increase of 0.96 (1.54) to 1.03 (1.42) for IGHD patients born SGA.

**Table 2:** GH response outcomes over time by year after enrollment and subpopulation^a^.

	SGA, peak GH ≥10 ng/mL			SGA (non-IGHD), peak GH <10 ng/mL			IGHD born SGA			IGHD not born SGA			ISS		
	**Year 1**	**Year 2**	**Year 3**	**Year 1**	**Year 2**	**Year 3**	**Year 1**	**Year 2**	**Year 3**	**Year 1**	**Year 2**	**Year 3**	**Year 1**	**Year 2**	**Year 3**
*n*	28	28	28	15	15	15	86	86	86	709	709	709	44	44	44
IGF-I SDS^b^, *n*	20	14	15	3	7	8	49	57	51	387	415	400	23	26	20
Mean (s.d.)	0.68 (2.17)	1.13 (1.69)	1.40 (1.14)	−0.11 (1.46)	0.38 (1.79)	1.19 (2.29)	0.96 (1.54)	0.96 (1.49)	1.03 (1.42)	0.19 (1.69)	0.80 (1.51)	1.00 (1.54)	0.21 (1.93)	0.58 (0.99)	0.40 (1.29)
BA/CA, *n*	11	7	17	7	8	9	47	57	61	335	471	437	22	24	20
Mean (s.d.)	0.78 (0.16)	0.87 (0.11)	0.91 (0.14)	0.83 (0.11)	0.88 (0.11)	0.88 (0.13)	0.88 (0.17)	0.88 (0.14)	0.92 (0.12)	0.87 (0.13)	0.89 (0.12)	0.91 (0.10)	0.86 (0.13)	0.88 (0.11)	0.91 (0.10)
BMI SDS^b^, *n*	27	26	28	12	13	13	80	79	79	635	660	664	41	41	42
Mean (s.d.)	−0.90 (1.43)	−0.90 (1.18)	−0.57 (1.06)	−0.85 (2.28)	−0.34 (1.22)	−0.21 (1.11)	−0.43 (1.16)	−0.34 (1.14)	−0.28 (1.03)	−0.18 (1.10)	−0.10 (1.13)	0.02 (1.07)	−0.25 (0.95)	−0.19 (0.93)	−0.13 (0.95)
Height SDS^b^, *n*	27	26	28	12	13	13	80	79	79	635	659	663	41	41	42
Mean (s.d.)	−2.43 (0.71)	−1.97 (0.77)	−1.63 (0.73)	−1.89 (0.60)	−1.45 (0.71)	−1.18 (0.79)	−2.08 (0.69)	−1.63 (0.72)	−1.34 (0.72)	−1.86 (0.78)	−1.40 (0.78)	−1.04 (0.80)	−1.82 (0.48)	−1.62 (0.60)	−1.34 (0.59)
GH dose, *n*	26	26	28	12	13	13	75	72	74	599	629	631	31	32	33
Mean, mg/kg/day (s.d.)	0.05 (0.01)	0.05 (0.01)	0.05 (0.02)	0.05 (0.02)	0.05 (0.02)	0.05 (0.02)	0.04 (0.01)	0.05 (0.01)	0.05 (0.01)	0.05 (0.01)	0.05 (0.01)	0.05 (0.02)	0.05 (0.01)	0.05 (0.01)	0.05 (0.01)

^a^Values shown are mean SDS for each year after enrollment; ^b^higher mean SDS indicates better outcomes.

BA/CA, bone age/chronological age; BMI, body mass index; GH, growth hormone; IGF-I, insulin-like growth factor 1; IGHD, isolated growth hormone deficiency; ISS, idiopathic short stature; s.d., standard deviation; SDS, standard deviation score; SGA, small for gestational age.

### Change from baseline in GH response and treatment by year after enrollment and subpopulation

**Figure 1:**
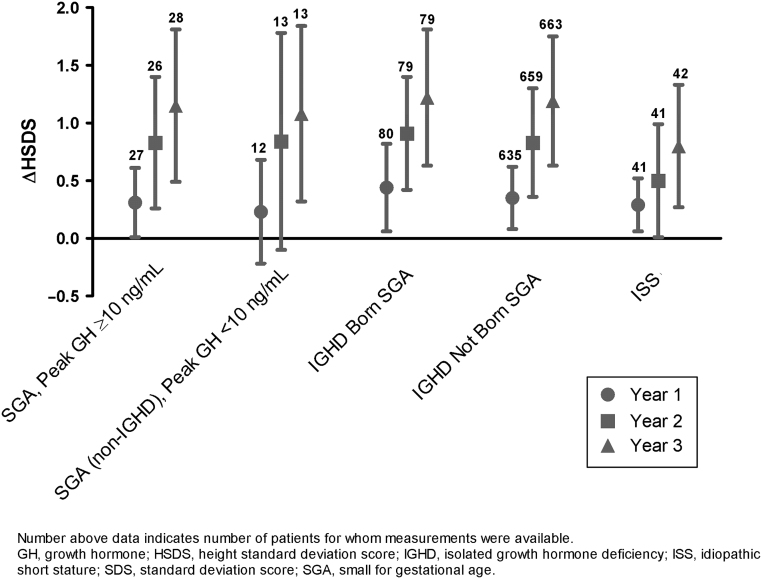
Mean and SDS change from baseline HSDS over time.

Consistent with these response data, mean ΔHSDS relative to baseline levels progressively increased over each year of follow-up for each patient subpopulation (Fig. 1). Increases in mean ΔHSDS were similar between SGA patients with GH deficiency (0.23 in year 1 to 1.08 in year 3) and patients born SGA and GH sufficient (0.31 in year 1 to 1.15 in year 3). Patients with IGHD not born SGA had a greater increase in mean ΔHSDS (0.35 in year 1 to 1.19 in year 3) than patients with IGHD born SGA (0.44 in year 1 to 1.22 in year 3). Patients with ISS had the lowest mean ΔHSDS of all subgroups at year 3.

### Differences by subpopulation in changes in HSDS from baseline: repeated measurements mixed model results

To control for potential covariates influencing ΔHSDS results, differences were also compared by ANCOVA and adjusted for year of follow-up, gender, baseline age and baseline HSDS, with an estimated difference from baseline of 0.5855 (Table 3). Significant differences in ∆HSDS between subpopulations remained after adjusting for each of these variables. Additional estimates quantified differences in ∆HSDS compared with a specific reference population (ie, the IGHD not born SGA group, which had the greatest ∆HSDS during GH treatment). Compared to the IGHD not born SGA population, individuals with ISS and those born SGA with peak GH ≥10 ng/mL or ≥20 mIU/L also showed significant improvements in ∆HSDS with GH treatment. Overall, the study population showed a significant improvement in ∆HSDS, measured as yearly change over baseline, by 0.8070 s.d. in year 3 over year 1. A significant decline in ∆HSDS by approximately 0.059 was estimated per each year of delay in start of treatment. For each unit of increase in baseline HSDS, improvement from GH treatment declined by an estimated 0.1275 ΔHSDS.

**Table 3:** Differences in ΔHSDS from baseline – repeated measurements mixed model results.

Fixed effect	Estimate	95% CI LB	95% CI UB	*T*-test *P* value
Intercept	0.5855	0.4489	0.7221	
Subpopulation indicators^a^				<0.0001^b^
IGHD not born SGA (ref)	–	–	–	–
SGA, peak GH ≥10 ng/mL	−0.2419	−0.3842	−0.09961	0.0009
SGA (non-IGHD), peak GH <10 ng/mL	−0.1568	−0.3513	0.03766	0.1139
IGHD born SGA	−0.04442	−0.1301	0.04121	0.3089
ISS	−0.2463	−0.3596	−0.1331	<0.0001
Year indicators				<0.0001^b^
Year 1 (ref)	–	–	–	–
Year 2	0.4553	0.4312	0.4793	<0.0001
Year 3	0.8070	0.7829	0.8311	<0.0001
Gender				
Female (ref)	–	–	–	–
Male	0.1062	0.0427	0.1697	0.0011
Age at baseline	−0.05898	−0.0677	−0.05026	<0.0001
HSDS at baseline	−0.1275	−0.1612	−0.09376	<0.0001

*n* = 2438 visits by 866 patients.

^a^Adjusted for year of follow-up, gender, baseline age, and HSDS; ^b^overall *F*-test *P* value.

GH, growth hormone; HSDS, height standard deviation score; IGHD, isolated growth hormone deficiency; ISS, idiopathic short stature; LB, lower bound; ref, reference; SGA, small for gestational age; UB, upper bound.

### Differences by subpopulation in changes in IGF-I SDS from baseline: repeated measurements mixed model results

ANCOVA was similarly used to estimate differences in ΔIGF-I SDS while adjusting for year of follow-up, gender, baseline age and baseline HSDS. After controlling for each of these variables, significant differences were observed between subpopulations in ∆IGF-I SDS (Table 4). The GH-sufficient SGA subpopulation showed the greatest improvement from baseline in ΔIGF-I SDS (estimated 0.269 compared with the reference subpopulation (IGHD not born SGA)). In comparison, the GH-deficient SGA population had an estimated ΔIGF-I SDS improvement of −0.305. In the overall study population, ΔIGF-I SDS compared with year 1 improved significantly in year 2 (estimated 0.5443) and in year 3 (estimated 0.7387).

**Table 4:** Differences in ΔIGF-I SDS from baseline – repeated measurements mixed model results.

Fixed effect	Estimate	95% CI LB	95% CI UB	*T*-test *P* value
Intercept	0.7326	0.3607	1.1044	
Subpopulation indicators^a^				0.0208^b^
IGHD not born SGA (ref)	–	–	–	–
SGA, peak GH ≥10 ng/mL	0.269	−0.2715	0.8095	0.3289
SGA (non-IGHD), peak GH <10 ng/mL	−0.305	−1.1046	0.4946	0.4542
IGHD born SGA	0.103	−0.2124	0.4185	0.5215
ISS	−0.6673	−1.0982	−0.2365	0.0024
Year indicators				<0.0001^b^
Year 1 (ref)	–	–	–	–
Year 2	0.5443	0.4027	0.6858	<0.0001
Year 3	0.7387	0.5951	0.8823	<0.0001
Gender				
Female (ref)	–	–	–	–
Male	0.3211	0.08136	0.5609	0.0087
Age at baseline	−0.01875	−0.05362	0.01613	0.2916
IGF-I SDS at baseline	−0.6524	−0.7139	−0.591	<0.0001

*n* = 1495 visits by 712 patients.

^a^Adjusted for year of follow-up, gender, baseline age, and HSDS; ^b^overall *F*-test *P* value.

GH, growth hormone; IGF-I, insulin-like growth factor 1; IGHD, isolated growth hormone deficiency; ISS, idiopathic short stature; LB, lower bound; ref, reference; SDS, standard deviation score; SGA, small for gestational age; UB, upper bound.

### Mixed models LS means for ∆HSDS and ∆IGF-I SDS at year 3 by subpopulation

Estimates of ∆HSDS and ΔIGF-I SDS at year 3 were generated as LS means by assigning mean values for all other covariates (male, 79%; baseline age, 9.6 years; enrollment HSDS, −2.27; enrollment IGF-I SDS, −1.57) (Table 5). The estimate for ∆HSDS at year 3 was higher for patients born SGA with GH deficiency, with an LS mean of 1.0575, compared to patients born SGA who were GH sufficient (LS mean, 0.9713). Conversely, the estimate for ∆IGF-I SDS at year 3 was higher for patients born SGA who were GH sufficient (LS mean, 2.8806) compared to patients born SGA who were GH deficient (LS mean, 2.3263). For IGHD patients, those born SGA had a lower estimated ∆HSDS at year 3 (LS mean, 1.1693) compared to IGHD patients who were not born SGA (LS mean, 1.2142). Estimated ∆IGF-I SDS was higher for patients with IGHD born SGA (LS mean, 2.7341) compared to IGHD patients not born SGA (LS mean, 2.6273).

**Table 5:** Mixed models least-squares means for ∆HSDS and ∆IGF-I SDS at year 3.

Pairwise comparison	∆HSDS			∆IGF-I SDS		
	**LS meana**	**95% CI LB**	**95% CI UB**	**LS meana**	**95% CI LB**	**95% CI UB**
SGA, peak GH ≥10 ng/mL	0.9713	0.8316	1.1109	2.8806	2.3452	3.4161
SGA (non-IGHD), peak GH <10 ng/mL	1.0575	0.8649	1.2501	2.3263	1.5323	3.1202
IGHD born SGA	1.1693	1.0878	1.2508	2.7341	2.43	3.0382
IGHD not born SGA	1.2142	1.1841	1.2444	2.6273	2.5004	2.7543
ISS	0.9676	0.857	1.0781	1.9685	1.5451	2.392

Mixed model results in Tables 3 and 4.

^a^Assigned mean values for all other covariates: male = 79%, enrollment age = 9.6 years, enrollment HSDS = −2.27, enrollment IGF-I SDS = −1.57.

GH, growth hormone; HSDS, height standard deviation score; IGF-1, insulin-like growth factor 1; IGHD, isolated growth hormone deficiency; ISS, idiopathic short stature; LB, lower bound; LS, least squares; SGA, small for gestational age; UB, upper bound.

## Discussion

This analysis of data from the ANSWER Program demonstrates that treatment with GH resulted in improved growth outcomes for children with IGHD or SGA (GH sufficient or deficient) and ISS. With few exceptions, improvements were shown in all outcomes (IGF-I SDS, BMI SDS and HSDS) for all population groups over 3 years of treatment. Increases in ∆HSDS over time are consistent with results reported in other ANSWER publications (9, 13, 26). Previous publications have evaluated the effects of gender (26), age (9, 26), diagnosis (6) and baseline auxological characteristics (BMI, HSDS, IGF-I) (13) on responses to GH therapy; however, effects of birth size independent of GH status have not previously been assessed. In patients born SGA, effect of GH status (deficiency or sufficiency) on response to GH therapy is also unknown.

Improvements in mean HSDS were observed for all patient subpopulations, indicating the benefits of GH treatment. In the analysis presented here, two subpopulations of patients with GHD who were born SGA were compared: those who were primarily diagnosed with SGA and found to be GH insufficient and those who were diagnosed with IGHD and whose historical auxological data fulfilled criteria for SGA. The SGA group who were GH insufficient showed a greater improvement in HSDS than patients with IGHD born SGA. Previous reports of data from the ANSWER Program indicated that increases in HSDS were similar between patients born SGA and those with IGHD (6, 7). The analyses in those studies, however, did not consider GH sufficiency among patients born SGA. The data in this report demonstrate the variability in responses to GH treatment among patients born SGA depending on GH status. For example, increases in mean ∆HSDS were greater for patients born SGA who were GH sufficient than for patients born SGA who were GH insufficient. However, increases in ∆BMI SDS from a negative BMI SDS, which is considered a positive response to GH therapy and indicates that the patient’s BMI is approaching the normal range, were greater for patients born SGA who were GH insufficient than for those who were GH sufficient. In the IGHD group, GH responses also appeared to be affected by SGA status, as patients with IGHD not born SGA showed a greater increase in mean ∆HSDS than patients with IGHD born SGA. Given the variability in response to GH therapy among SGA patients, GH status should be considered when setting treatment goals. Regarding the ISS cohort, while mean daily GH dose was higher for this patient group (0.06 mg/kg/day vs 0.05 mg/kg/day for all other groups), these patients appeared to benefit the least from GH therapy (eg, change in HSDS and IGF-I from year 1 to year 3 was the least for the ISS cohort). It is important to note that of the cohorts analyzed in this study, patients with ISS may be the most heterogeneous, as their causes of short stature may be variable. Etiologies may include GH resistance/diminished responsiveness so that larger dosages are necessary for clinical responsiveness. Given this likelihood, it is recognized that growth responses based upon similar dosages cannot be compared meaningfully.

In addition to HSDS, mean IGF-I SDS also increased in most patient groups while GH dose remained constant over the 3-year period in all groups. Although the IGHD born SGA subpopulation demonstrated similar changes in HSDS to the other groups, mean IGF-I SDS only increased from 0.96 to 1.03. In comparison, the other groups with GH insufficiency demonstrated greater increases in IGF-I SDS; the IGHD not born SGA group increased from 0.19 in year 1 to 1.00 in year 3, and SGA with GH deficiency group increased from −0.11 to 1.19; the SGA and GH sufficient group also showed a greater increase in IGF-I SDS, from 0.68 in year 1 to 1.40 in year 3.

The repeated measurements model for ∆HSDS derived from this analysis indicates that older baseline age and having a lower difference in HSDS at baseline may reduce the effect of GH treatment on HSDS. The model is consistent with findings from other analyses on data from the ANSWER Program that included patients with GHD, SGA and ISS, which demonstrated that younger age at baseline and lower baseline HSDS were factors that were associated with higher ∆HSDS after approximately 4 years of GH treatment (7, 13). This result reiterates the importance of initiating GH therapy at a young age, which has been emphasized before.

There are several potential limitations of this study. Given that the ANSWER Program is an observational study, there may be variations in data collection due to the large number of investigators who participated. In addition, it was at the discretion of the reporting physician to provide their patient’s diagnosis, and thus, variability among physicians’ diagnoses contribute to a certain lack of uniformity. There is also potential for information bias due to missing or erroneous data points resulting from misdiagnosis or failure to report confounding variables. Differences in laboratory assays (eg, IGF-I assays, GH assays) and diagnostic procedures among sites reporting information may affect outcomes, and considering the variability in IGF-I assays, this especially limits interpretation of IGF-I data. Another potential limitation is the differences in availability of diagnostic technologies among clinics; for this reason, some patients may be missing results for some tests, such as the GH stimulation test. Further, as noted earlier, there are situations of GH resistance that may occur within the categorical diagnoses of both ISS and SGA that are difficult to diagnose or that are yet to be described.

In conclusion, results of this analysis demonstrate that the greatest benefits of GH treatment were shown for patients diagnosed with IGHD who were not born SGA, those diagnosed with IGHD who were born SGA and those born SGA who were GH deficient. Of note, among patients with IGHD, birth size appears to affect response to GH therapy, as IGHD patients who were not born SGA showed greater improvements in growth-related outcomes compared to IGHD patients who were born SGA. Regarding patients born SGA, GH responses were variable between those who were GH sufficient and those who were GH deficient. While the assessment of GH status in patients born SGA has not been recommended in the literature or by existing guidelines, the results reported here suggest that determining GH status may help explain some of the variation in response to GH therapy in children born SGA. Therefore, GH status may be yet another consideration for GH therapy optimization in these patients. Further studies, including genetic analyses, are needed to elucidate additional aspects of SGA that impact growth.

## Declaration of interest

R Rapaport has served as a consultant for Novo Nordisk Inc. and Sandoz. P Lee has served on advisory boards for Abbvie and Novo Nordisk, speakers bureau for AbbVie and is on the safety monitoring committee for Versartis. J Ross has served as a consultant for Novo Nordisk and Opko and has research supported by Versartis and Novo Nordisk. P Saenger has nothing to declare. V Ostrow is an employee of Novo Nordisk Inc. G Piccoli is an employee of Novo Nordisk Inc.
